# Cross-project HTS-datamining

**DOI:** 10.1186/1758-2946-3-S1-O2

**Published:** 2011-04-19

**Authors:** Wolfgang Guba, Daniel Stoffler

**Affiliations:** 1 F. Hoffmann-La Roche Ltd., Basel, CH-4070, Switzerland

## 

Over the years a massive amount of high-throughput screening (HTS) data has been collected, however, the data are mainly utilized for providing lead generation programs with chemical entry points. The comparison of molecular structures and HTS data across many projects allows to identify and validate structural patterns of frequent hitters, i.e. compounds which generate multiple hits in various target families. The identification of frequent hitters is an important component in maintaining a high-quality screening deck and supports project teams in the triaging of HTS hit lists. In addition, frequent hitters will be contrasted with privileged motifs which are believed to show activities in specific target classes only. The talk will also address the question what causes compounds to be frequent hitters, and in-silico prediction methods will be discussed.

